# Crystal structure of di­benzyl­dimethyl­silane

**DOI:** 10.1107/S2056989015008713

**Published:** 2015-05-09

**Authors:** Lena Knauer, Christopher Golz, Ulrike Kroesen, Stephan G. Koller, Carsten Strohmann

**Affiliations:** a Fakultät für Chemie und Chemische Biologie, Technische Universität Dortmund, Otto-Hahn-Strasse 6, 44227 Dortmund, Germany

**Keywords:** crystal structure, di­benzyl­dimethyl­silane, Bent’s rule

## Abstract

In the title compound, C_16_H_20_Si, a geometry different from an ideal tetra­hedron can be observed at the Si atom. The bonds from Si to the benzylic C atoms [Si—C = 1.884 (1) and 1.883 (1) Å] are slightly elongated compared to the Si—C_meth­yl_ bonds [Si—C = 1.856 (1) and 1.853 (1) Å]. The C_benz­yl_—Si—C_benz­yl_ bond angle [C—Si—C = 107.60 (6)°] is decreased from the ideal tetra­hedral angle by 1.9°. These distortions can be explained easily by Bent’s rule. In the crystal, mol­ecules inter­act only by van der Waals forces.

## Related literature   

The chemistry of silicon exhibits several differences compared to carbon, its lighter congener. Being a representative of the third period, the silicon atom provides deviant reactivity and structural features including the formation of penta­valent inter­mediates (Chuit *et al.*, 1993 ▸; Cypryk & Apeloig, 2002 ▸) as well as silicon-specific effects like the α- or β-effect (Whitmore & Sommer, 1946 ▸; Sommer & Whitmore, 1946 ▸). For the correlation of bond lengths and angles with the electronegativity of substituents, see: Bent (1961 ▸) and for the same effect in the related compound MePh_2_SiBn, see: Koller *et al.* (2015 ▸). For the reaction of silyllithium reagents to benzyl­silanes, see: Strohmann *et al.* (2004 ▸). For the α-li­thia­tion of methyl­silanes, see: Däschlein *et al.* (2010 ▸). For the structure and reactivity of α-li­thia­ted benzyl­silanes, see: Ott *et al.* (2008 ▸), Strohmann *et al.* (2002 ▸).
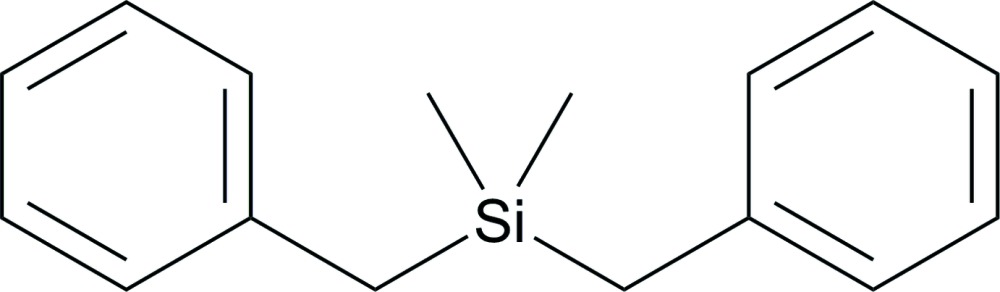


## Experimental   

### Crystal data  

C_16_H_20_Si
*M* *_r_* = 240.41
Monoclinic, 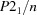

*a* = 6.1045 (2) Å
*b* = 19.8512 (6) Å
*c* = 11.8396 (3) Å
β = 98.069 (3)°
*V* = 1420.54 (7) Å^3^
*Z* = 4
Mo *K*α radiation
μ = 0.14 mm^−1^
*T* = 173 K
0.2 × 0.1 × 0.1 mm

### Data collection  

Oxford Diffraction Xcalibur, Sapphire3 diffractometer
Absorption correction: multi-scan (*CrysAlis PRO*; Oxford Diffraction, 2010 ▸) *T*_min_ = 0.940, *T*_max_ = 1.000
21539 measured reflections
2800 independent reflections
2280 reflections with *I* > 2σ(*I*)
*R*_int_ = 0.035

### Refinement  

*R*[*F* ^2^ > 2σ(*F* ^2^)] = 0.033
*wR*(*F* ^2^) = 0.088
*S* = 1.06
2800 reflections
156 parameters
H-atom parameters constrained
Δρ_max_ = 0.29 e Å^−3^
Δρ_min_ = −0.24 e Å^−3^

### 

Data collection: *CrysAlis PRO* (Oxford Diffraction, 2010 ▸); cell refinement: *CrysAlis PRO*; data reduction: *CrysAlis PRO*; program(s) used to solve structure: *SHELXS97* (Sheldrick, 2008 ▸); program(s) used to refine structure: *SHELXL2014* (Sheldrick, 2015 ▸); molecular graphics: *OLEX2* (Dolomanov *et al.*, 2009 ▸); software used to prepare material for publication: *OLEX2*.

## Supplementary Material

Crystal structure: contains datablock(s) I. DOI: 10.1107/S2056989015008713/fk2087sup1.cif

Structure factors: contains datablock(s) I. DOI: 10.1107/S2056989015008713/fk2087Isup2.hkl

Supporting information file. DOI: 10.1107/S2056989015008713/fk2087Isup3.cml

**Fig. 1.**
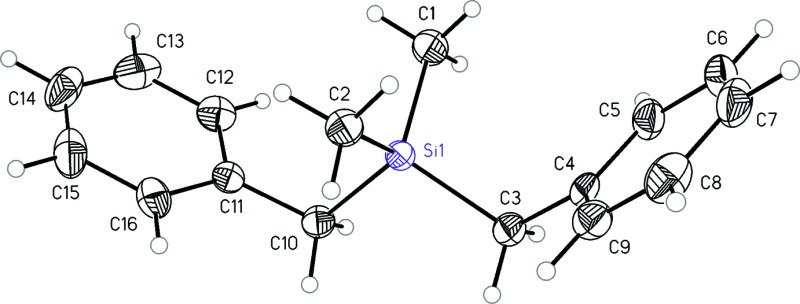
Molecular structure of the title compound with anisotropic displacement ellipsoids drawn at 50% probability level.

. DOI: 10.1107/S2056989015008713/fk2087fig1.tif
Mol­ecular structure of the title compound with anisotropic displacement ellipsoids drawn at 50% probability level.

CCDC reference: 1063257

Additional supporting information:  crystallographic information; 3D view; checkCIF report

## supplementary crystallographic information

### S1. Structural commentary

The chemistry of silicon exhibits several differences compared to carbon, its lighter congener. Being a representative of the third period, the silicon atom provides deviant reactivity and structural features. Among others, the formation of penta­valent inter­mediates (Chuit *et al.*, 1993; Cypryk & Apeloig, 2002) as well as silicon-specific effects like the α- or β-effect (Whitmore & Sommer, 1946; Sommer & Whitmore, 1946) can be listed. Last but not least, the low electronegativity of silicon compared to carbon is a feature that has to be considered.

In the title compound, two different types of Si–C bonds can be observed. Comparing the Si–C_methyl_ bonds Si1–C1 [1.856 (1) Å] and Si1–C2 [1.853 (1) Å] to the Si–C_benzyl_ bonds Si1–C3 [1.884 (1) Å] and Si1–C10 [1.883 (1) Å], a difference of 0.03 Å becomes obvious. This divergence can be explained by Bent's rule (Bent, 1961): atomic s-character is concentrated in orbitals forming bonds with electropositive substituents. In return, the orbitals of bonds with electronegative substituents are featured by a high p-character, thus leading to elongated bond lengths and bond angles shifted towards 90°. In the title compound, the carbon atoms directly bonded to the silicon center exhibit unequal electronegativities. Due to the ability of benzylic carbon atoms to stabilize a negative charge, they are of higher electronegativity than carbon atoms of methyl groups. According to Bent's rule, atomic p-character is concentrated in the orbitals forming the Si–C_benzyl_ bonds to a higher level than in the Si–C_methyl_ bonds. This assumption is furthermore affirmed by the C—Si—C bond angles observed in the title compound. The bond angle between the methyl carbon atoms is very close to the ideal tetra­hedral angle [C1–Si1–C2 109.89 (7)°], the angle between the benzyl carbon atoms is slightly smaller [C3–Si1–C10 107.60 (6)°], as it would be expected for bonds formed by orbitals with increased p-character.

The same effect can be observed in the related compound MePh_2_SiBn (Koller *et al.*, 2015). According to the title compound, the Si–C_methyl_ bond is the shortest [1.853 (1) Å], and the Si–C_benzyl_ bond is the longest [1.876 (2) Å]. The Si–C_phenyl_ bonds are settled in between at 1.873 (1) Å and 1.869 (1) Å, respectively.

### S2. Refinement

Crystal data, data collection and structure refinement details are summarized in Table 1.

Hydrogen atoms were located from difference Fourier maps, refined at idealized positions riding on the carbon atoms with isotropic displacement parameters U_iso_(H) = 1.2U_eq_(C) or 1.5U_eq_(-CH_3_) and C–H = 0.95-0.99 Å. All CH_3_ hydrogen atoms were allowed to rotate but not to tip.

### Crystal data

**Table:**

C_16_H_20_Si	*F*(000) = 520
*M**_r_* = 240.41	*D*_x_ = 1.124 Mg m^−^^3^
Monoclinic, *P*2_1_/*n*	Mo *K*α radiation, λ = 0.71073 Å
*a* = 6.1045 (2) Å	Cell parameters from 10295 reflections
*b* = 19.8512 (6) Å	θ = 2.7–29.2°
*c* = 11.8396 (3) Å	µ = 0.14 mm^−^^1^
β = 98.069 (3)°	*T* = 173 K
*V* = 1420.54 (7) Å^3^	Block, colourless
*Z* = 4	0.2 × 0.1 × 0.1 mm

### Data collection

**Table:**

Oxford Diffraction Xcalibur, Sapphire3 diffractometer	2800 independent reflections
Radiation source: Enhance (Mo) X-ray Source	2280 reflections with *I* > 2σ(*I*)
Graphite monochromator	*R*_int_ = 0.035
Detector resolution: 16.0560 pixels mm^-1^	θ_max_ = 26.0°, θ_min_ = 2.7°
ω scans	*h* = −7→7
Absorption correction: multi-scan (*CrysAlis PRO*; Oxford Diffraction, 2010)	*k* = −24→24
*T*_min_ = 0.940, *T*_max_ = 1.000	*l* = −14→14
21539 measured reflections	

### Refinement

**Table:**

Refinement on *F*^2^	0 restraints
Least-squares matrix: full	Hydrogen site location: inferred from neighbouring sites
*R*[*F*^2^ > 2σ(*F*^2^)] = 0.033	H-atom parameters constrained
*wR*(*F*^2^) = 0.088	*w* = 1/[σ^2^(*F*_o_^2^) + (0.0528*P*)^2^ + 0.0485*P*] where *P* = (*F*_o_^2^ + 2*F*_c_^2^)/3
*S* = 1.06	(Δ/σ)_max_ < 0.001
2800 reflections	Δρ_max_ = 0.29 e Å^−^^3^
156 parameters	Δρ_min_ = −0.24 e Å^−^^3^

### Special details

**Table:**

Experimental. CrysAlisPro, Oxford Diffraction Ltd., Version 1.171.33.55 (release 05-01-2010 CrysAlis171 .NET) (compiled Jan 5 2010,16:28:46) Empirical absorption correction using spherical harmonics, implemented in SCALE3 ABSPACK scaling algorithm.
Geometry. All e.s.d.'s (except the e.s.d. in the dihedral angle between two l.s. planes) are estimated using the full covariance matrix. The cell e.s.d.'s are taken into account individually in the estimation of e.s.d.'s in distances, angles and torsion angles; correlations between e.s.d.'s in cell parameters are only used when they are defined by crystal symmetry. An approximate (isotropic) treatment of cell e.s.d.'s is used for estimating e.s.d.'s involving l.s. planes.

### Fractional atomic coordinates and isotropic or equivalent isotropic displacement parameters (Å^2^)

**Table:**

	*x*	*y*	*z*	*U*_iso_*/*U*_eq_	
Si1	0.13789 (6)	0.14013 (2)	0.03688 (3)	0.01945 (12)	
C11	0.1208 (2)	0.10937 (7)	−0.19747 (11)	0.0221 (3)	
C9	0.4409 (2)	0.16077 (7)	0.32428 (12)	0.0286 (3)	
H9	0.5690	0.1513	0.2898	0.034*	
C12	−0.0953 (2)	0.10519 (8)	−0.25332 (12)	0.0301 (3)	
H12	−0.2001	0.1387	−0.2402	0.036*	
C16	0.2691 (2)	0.05980 (7)	−0.22010 (12)	0.0299 (3)	
H16	0.4177	0.0616	−0.1833	0.036*	
C5	0.0758 (2)	0.20416 (7)	0.31346 (12)	0.0284 (3)	
H5	−0.0500	0.2252	0.2716	0.034*	
C3	0.2631 (2)	0.20813 (7)	0.13668 (11)	0.0243 (3)	
H3A	0.4179	0.2157	0.1238	0.029*	
H3B	0.1805	0.2506	0.1185	0.029*	
C10	0.1896 (2)	0.16338 (7)	−0.11131 (11)	0.0243 (3)	
H10A	0.1076	0.2052	−0.1350	0.029*	
H10B	0.3493	0.1727	−0.1100	0.029*	
C4	0.2605 (2)	0.19144 (7)	0.26040 (11)	0.0220 (3)	
C8	0.4376 (3)	0.14376 (8)	0.43742 (13)	0.0368 (4)	
H8	0.5636	0.1233	0.4800	0.044*	
C2	0.2653 (2)	0.05739 (7)	0.07812 (12)	0.0263 (3)	
H2A	0.2290	0.0443	0.1531	0.039*	
H2B	0.4263	0.0607	0.0816	0.039*	
H2C	0.2081	0.0234	0.0215	0.039*	
C1	−0.1645 (2)	0.13650 (8)	0.04158 (13)	0.0306 (3)	
H1A	−0.2328	0.1042	−0.0153	0.046*	
H1B	−0.2293	0.1812	0.0248	0.046*	
H1C	−0.1912	0.1222	0.1177	0.046*	
C6	0.0720 (3)	0.18677 (8)	0.42632 (13)	0.0356 (4)	
H6	−0.0561	0.1959	0.4610	0.043*	
C13	−0.1588 (3)	0.05285 (9)	−0.32761 (12)	0.0401 (4)	
H13	−0.3072	0.0506	−0.3646	0.048*	
C14	−0.0101 (3)	0.00404 (9)	−0.34877 (12)	0.0444 (5)	
H14	−0.0549	−0.0320	−0.3997	0.053*	
C7	0.2521 (3)	0.15639 (8)	0.48868 (13)	0.0378 (4)	
H7	0.2489	0.1442	0.5660	0.045*	
C15	0.2045 (3)	0.00808 (8)	−0.29506 (13)	0.0414 (4)	
H15	0.3090	−0.0251	−0.3098	0.050*	

### Atomic displacement parameters (Å^2^)

**Table:**

	*U*^11^	*U*^22^	*U*^33^	*U*^12^	*U*^13^	*U*^23^
Si1	0.0205 (2)	0.0177 (2)	0.0207 (2)	−0.00025 (15)	0.00477 (14)	−0.00023 (15)
C11	0.0291 (7)	0.0214 (7)	0.0167 (6)	−0.0014 (6)	0.0054 (5)	0.0054 (5)
C9	0.0277 (8)	0.0259 (8)	0.0318 (8)	−0.0007 (6)	0.0030 (6)	−0.0024 (6)
C12	0.0329 (8)	0.0329 (9)	0.0239 (7)	0.0003 (7)	0.0021 (6)	0.0089 (6)
C16	0.0351 (8)	0.0315 (8)	0.0240 (7)	0.0050 (7)	0.0076 (6)	0.0017 (6)
C5	0.0306 (8)	0.0268 (8)	0.0281 (7)	0.0025 (6)	0.0051 (6)	−0.0074 (6)
C3	0.0268 (7)	0.0211 (7)	0.0251 (7)	−0.0015 (6)	0.0047 (6)	−0.0010 (6)
C10	0.0288 (8)	0.0201 (7)	0.0245 (7)	−0.0011 (6)	0.0054 (6)	0.0024 (6)
C4	0.0264 (7)	0.0162 (7)	0.0233 (7)	−0.0040 (5)	0.0032 (6)	−0.0054 (5)
C8	0.0434 (9)	0.0314 (9)	0.0324 (8)	−0.0012 (7)	−0.0063 (7)	0.0023 (7)
C2	0.0294 (8)	0.0221 (8)	0.0277 (7)	0.0009 (6)	0.0054 (6)	0.0026 (6)
C1	0.0244 (7)	0.0329 (9)	0.0352 (8)	0.0005 (6)	0.0062 (6)	−0.0046 (7)
C6	0.0447 (9)	0.0331 (9)	0.0320 (8)	−0.0048 (7)	0.0156 (7)	−0.0109 (7)
C13	0.0446 (9)	0.0497 (11)	0.0230 (8)	−0.0177 (8)	−0.0053 (7)	0.0082 (7)
C14	0.0786 (13)	0.0339 (9)	0.0212 (7)	−0.0190 (9)	0.0084 (8)	−0.0050 (7)
C7	0.0630 (11)	0.0301 (9)	0.0206 (7)	−0.0107 (8)	0.0067 (7)	−0.0029 (6)
C15	0.0659 (12)	0.0296 (9)	0.0318 (8)	0.0052 (8)	0.0175 (8)	−0.0025 (7)

### Geometric parameters (Å, º)

**Table:**

Si1—C3	1.8838 (14)	C3—C4	1.5040 (18)
Si1—C10	1.8832 (13)	C10—H10A	0.9900
Si1—C2	1.8534 (14)	C10—H10B	0.9900
Si1—C1	1.8563 (14)	C8—H8	0.9500
C11—C12	1.3928 (19)	C8—C7	1.381 (2)
C11—C16	1.3887 (19)	C2—H2A	0.9800
C11—C10	1.4988 (19)	C2—H2B	0.9800
C9—H9	0.9500	C2—H2C	0.9800
C9—C4	1.3861 (19)	C1—H1A	0.9800
C9—C8	1.384 (2)	C1—H1B	0.9800
C12—H12	0.9500	C1—H1C	0.9800
C12—C13	1.381 (2)	C6—H6	0.9500
C16—H16	0.9500	C6—C7	1.375 (2)
C16—C15	1.378 (2)	C13—H13	0.9500
C5—H5	0.9500	C13—C14	1.375 (2)
C5—C4	1.3888 (19)	C14—H14	0.9500
C5—C6	1.383 (2)	C14—C15	1.376 (2)
C3—H3A	0.9900	C7—H7	0.9500
C3—H3B	0.9900	C15—H15	0.9500
			
C10—Si1—C3	107.60 (6)	C9—C4—C5	117.79 (13)
C2—Si1—C3	110.57 (6)	C9—C4—C3	120.81 (12)
C2—Si1—C10	110.09 (6)	C5—C4—C3	121.37 (12)
C2—Si1—C1	109.89 (7)	C9—C8—H8	119.8
C1—Si1—C3	109.07 (6)	C7—C8—C9	120.35 (15)
C1—Si1—C10	109.58 (6)	C7—C8—H8	119.8
C12—C11—C10	121.48 (13)	Si1—C2—H2A	109.5
C16—C11—C12	117.78 (13)	Si1—C2—H2B	109.5
C16—C11—C10	120.68 (12)	Si1—C2—H2C	109.5
C4—C9—H9	119.4	H2A—C2—H2B	109.5
C8—C9—H9	119.4	H2A—C2—H2C	109.5
C8—C9—C4	121.11 (14)	H2B—C2—H2C	109.5
C11—C12—H12	119.7	Si1—C1—H1A	109.5
C13—C12—C11	120.64 (15)	Si1—C1—H1B	109.5
C13—C12—H12	119.7	Si1—C1—H1C	109.5
C11—C16—H16	119.4	H1A—C1—H1B	109.5
C15—C16—C11	121.11 (14)	H1A—C1—H1C	109.5
C15—C16—H16	119.4	H1B—C1—H1C	109.5
C4—C5—H5	119.5	C5—C6—H6	119.7
C6—C5—H5	119.5	C7—C6—C5	120.51 (15)
C6—C5—C4	121.09 (14)	C7—C6—H6	119.7
Si1—C3—H3A	108.9	C12—C13—H13	119.6
Si1—C3—H3B	108.9	C14—C13—C12	120.86 (15)
H3A—C3—H3B	107.7	C14—C13—H13	119.6
C4—C3—Si1	113.22 (9)	C13—C14—H14	120.5
C4—C3—H3A	108.9	C13—C14—C15	119.01 (15)
C4—C3—H3B	108.9	C15—C14—H14	120.5
Si1—C10—H10A	109.0	C8—C7—H7	120.4
Si1—C10—H10B	109.0	C6—C7—C8	119.14 (14)
C11—C10—Si1	113.02 (9)	C6—C7—H7	120.4
C11—C10—H10A	109.0	C16—C15—H15	119.7
C11—C10—H10B	109.0	C14—C15—C16	120.60 (15)
H10A—C10—H10B	107.8	C14—C15—H15	119.7
			
Si1—C3—C4—C9	93.41 (13)	C10—C11—C12—C13	−176.30 (13)
Si1—C3—C4—C5	−84.53 (15)	C10—C11—C16—C15	176.82 (13)
C11—C12—C13—C14	−0.5 (2)	C4—C9—C8—C7	0.8 (2)
C11—C16—C15—C14	−0.5 (2)	C4—C5—C6—C7	0.0 (2)
C9—C8—C7—C6	−0.8 (2)	C8—C9—C4—C5	−0.4 (2)
C12—C11—C16—C15	−0.2 (2)	C8—C9—C4—C3	−178.39 (13)
C12—C11—C10—Si1	86.49 (14)	C2—Si1—C3—C4	−52.05 (11)
C12—C13—C14—C15	−0.3 (2)	C2—Si1—C10—C11	52.86 (11)
C16—C11—C12—C13	0.7 (2)	C1—Si1—C3—C4	68.89 (11)
C16—C11—C10—Si1	−90.42 (14)	C1—Si1—C10—C11	−68.10 (11)
C5—C6—C7—C8	0.4 (2)	C6—C5—C4—C9	0.0 (2)
C3—Si1—C10—C11	173.44 (9)	C6—C5—C4—C3	177.97 (13)
C10—Si1—C3—C4	−172.32 (9)	C13—C14—C15—C16	0.8 (2)
