# Supplementary figures and images for: Crystal structure of di­benzyl­dimethyl­silane
Source: Acta Crystallogr E Crystallogr Commun. 2015 May 9;71(Pt 6):o391–2. doi: 10.1107/S2056989015008713 (PMC4459354; doi:10.1107/S2056989015008713)

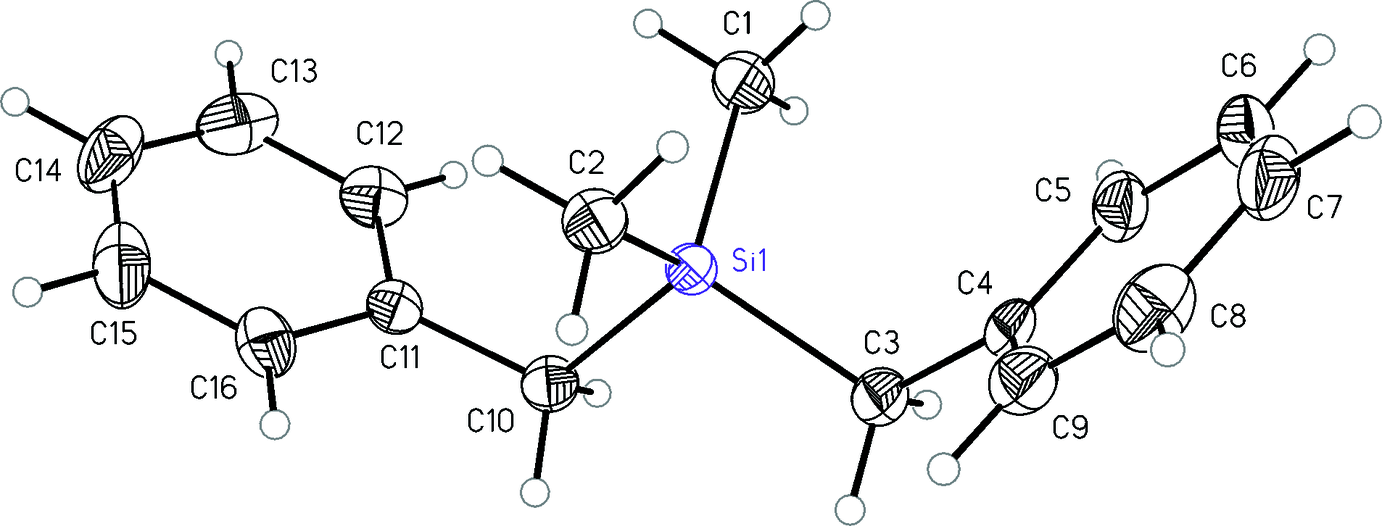

Supplement: Supplementary file 4 [file e-71-0o391-fig1.tif]
